# Mineral Fertilization Influences the Growth, Cryptolepine Yield, and Bioefficacy of *Cryptolepis sanguinolenta* (Lindl.) Schlt.

**DOI:** 10.3390/plants11010122

**Published:** 2022-01-01

**Authors:** Jacqueline Naalamle Amissah, Forgive Enyonam Alorvor, Benjamin Azu Okorley, Chris Mpere Asare, Dorcas Osei-Safo, Regina Appiah-Opong, Ivan Addae-Mensah

**Affiliations:** 1 Crop Science Department, University of Ghana, Legon, Accra P.O. Box LG 44, GM, Ghana; pe4give@gmail.com (F.E.A.); bazu_okorley@st.ug.edu.gh (B.A.O.); 2 Council for Scientific and Industrial Research (CSIR), Plant Genetic Resources Research Institute (PGRRI), Bunso P.O. Box 7, EE, Ghana; chrisasare83@gmail.com; 3 Chemistry Department, University of Ghana, Legon, Accra P.O. Box LG 56, GM, Ghana; dosei-safo@ug.edu.gh (D.O.-S.); iaddae-mensah@ug.edu.gh (I.A.-M.); 4 Department of Clinical Pathology, Noguchi Memorial Institute for Medical Research, College of Health Sciences, University of Ghana, Legon, Accra P.O. Box LG 581, GM, Ghana; rappiah-opong@noguchi.ug.edu.gh

**Keywords:** antiplasmodial, cytotoxicity, domestication, endangered medicinal plant, growth period, malaria

## Abstract

*Cryptolepis sanguinolenta* (Lindl.) Schlt., the main source of cryptolepine alkaloid, is intensively exploited in the wild to treat malaria and Lyme disease. In this study, the influence of four inorganic fertilizers (supplying N, P, K, or NPK) and four growth periods (3, 6, 9, and 12 months after transplanting) on the herb’s root biomass, cryptolepine content and yield, and biological activities were investigated in a pot and field trial. The results showed the application of N (in the form of Urea or NPK) increased root biomass yield, cryptolepine content, and cryptolepine yield compared to unfertilized plants. The 9-month-old plants recorded the maximum cryptolepine content (2.26 mg/100 mg dry root) and cryptolepine yield (304.08 mg/plant), indicating the perfect time to harvest the herb. Plant age at harvest had a more significant influence (50.6–55.7%) on cryptolepine production than fertilizer application (29.2–33.3%). Cryptolepine extracts from 9- to 12-month-old plants had the highest antiplasmodial activity (IC_50_ = 2.56–4.65 µg/mL) and drug selectivity index (2.15–3.91) against *Plasmodium falciparum* Dd2. These extracts were also cytotoxic to Jurkat leukaemia cell lines (CC_50_ < 62.56 µg/mL), indicating the possible use of cryptolepine for cancer management. Growing the herb in the field increased cryptolepine yield 2.5 times compared to growth in a pot, but this did not influence the antiplasmodial activity of the extract. Commercial cultivation of *C. sanguinolenta* for 9 months combined with N application could be a promising solution to the sustainable use of this threatened medicinal species.

## 1. Introduction

*Cryptolepis sanguinolenta* (Lindl.) Schlecter (family Periplocaceae) is among the indigenous herbal vines that have received much attention in West African traditional medicine. Its pharmacological activities are due to the predominant alkaloid cryptolepine along with the minor alkaloids (cryptoquindoline, quindoline, biscryptolepine, and cryptospirolepine) contained in the root of the plant [1,2,3,4]. In Ghana, *C. sanguinolenta* is widely used in decoctions and powders to treat malaria, a deadly disease of concern for public health, especially in areas where orthodox medicines are not readily available [5,6]. Currently, it is being studied in clinical trials as a potential therapy for the novel coronavirus disease 2019 (COVID-19) [7,8]. Elsewhere in the USA, the herb is used to treat Babesia, Lyme disease (*Borreliosis burgdorferi*), and Bartonella [9]. It is also appreciated for its antibacterial [10], anticancer [11,12], anti-diabetic [13], antifungal [14], and anti-inflammatory properties [15].

In recent years, Ghana’s herbal and brewery industries have seen an increase in demand for commercial products, including herbal teas, *Class Malacure*, *Herbaquine*, *Malaherb, and Phyto-Laria*, made from the roots of *C. sanguinolenta*. This demand has resulted in an excessive indulgence in harvesting from the wild, especially during the flowering period of the plant. This situation, coupled with climate change and land degradation, has drawn the attention of both ecologists and plant growers to the importance of protecting *C. sanguinolenta* from extinction [16]. There is an urgent need to champion domestication and cultivation protocols for the production of *C. sanguinolenta* to conserve the wild population and provide a sustainable source of plant material for use.

Earlier studies by Amissah et al. [17] found that the staking of *C. sanguinolenta* plants promoted pod formation and subsequent seed development. Additionally, protocols for the micropropagation of *C. sanguinolenta* have been developed to multiply planting materials [18]. However, the effects of environmental factors (for example, nutrient, climate, and water requirements) and the absence of reproducible bioactivity represent a major impediment in the cultivation of most medicinal plants [19,20]. Among these factors, plant nutrition has the greatest impact on plant growth and secondary metabolite yield, especially in marginal soils [21,22,23]. In recognition of the important role of plant nutrition, several studies have documented the nutritional requirements of other industrial and medicinal plants [23]. However, the nutrient demands and precise fertility targets of *C. sanguinolenta* as a commercial crop are unknown.

Furthermore, the economic value of *C. sanguinolenta* is determined by the cryptolepine content in the roots. Consequently, any rise in the cryptolepine levels provides a significant economic gain. Studies on Periwinkle (*Vinca minor*) and Datura (*Datura innoxia*) reported a rise in alkaloid content following the application of mineral NPK and N fertilizer, respectively [24,25]. Additionally, the use of fertilizers, notably those that provide nitrogen (N) and phosphorus (P), has been reported to improve the biomass of vegetative parts that produce secondary metabolites in aromatic medicinal plants (e.g., *Foeniculum vulgare*—fennel, *Cymbopogon martinii*—palmarosa, and *Ocimum basilicum*—basil cultivars) [26,27,28]. Therefore, the supply of mineral nutrients (N, P, or K) is expected to influence the production and accumulation of cryptolepine in *C. sanguinolenta*.

Amid efforts toward the cultivation and conservation of *C. sanguinolenta* in Ghana, the present study reports the comparative effects of four inorganic fertilizers (supplying either N, P, K, or combined NPK) and plant age on plant biomass accumulation, cryptolepine content, and yield. The bioactivity of cryptolepine extracts from plants of different ages was also determined on *Plasmodium* parasite and cancer cells. Such information will ultimately help understand the effect of inorganic fertilizers and plant growth periods on the commercial production of *C. sanguinolenta*.

## 2. Results and Discussion

### 2.1. Effect of Fertilizer Application on Vegetative Growth and Biomass Yield of C. sanguinolenta

To develop *C. sanguinolenta* into a profitable commercial crop, its agronomic performance needs to be improved. Therefore, in this study, the effect of four mineral fertilizers and four growth periods on the growth of *C. sanguinolenta* were investigated to provide information on the nutrients required to enhance its cultivation and productivity.

The data presented in Table 1 reveal that fertilizer treatments significantly influenced the vine number and girth in both the field and pot experiments. First, fertilizer P at 250 kg/ha and NPK at 262.5 kg/ha gave the highest number of vines (≈5) compared to the unfertilized controls in the field experiment. Additionally, all NPK treatments, except for NPK at 225 kg/ha, yielded the thickest vines for 3-, 6- and 12-month-old plants (3.97, 4.50, and 5.15 mm, respectively). Taking time into account, the application of NPK at 187.5 kg/ha alongside the 6-month growth period was the most effective for increasing vine girth and number, as these parameters in both experiments were consistently increased.

In both experiments, similar vine girths were obtained compared to the control (CK) plants at the different stages of growth. This indicated that plant age had little influence on the vine girth. In contrast, Amissah et al. [17] found a significant effect on plant girth between 9- and 12-month-old *C. sanguinolenta.*

Furthermore, fertilizer NPK at 225 kg/ha and N at 200 kg/ha produced significantly higher dry root biomass in the field experiment (22.68 and 22.40 g, respectively) (Table 2). Although the maximum root biomass yield was obtained in 6-month-old plants in the pot experiment, the results suggested that fertilizer treatments supplying N (N or NPK) improved the dry root biomass. Meanwhile, the aboveground biomass was increased by NPK at 265.2 kg/ha in the field experiment (111.68 g) and NPK at 187.5 kg/ha in the pot experiment (41.47 g)**.**

Overall, the growth response of *C. sanguinolenta* to K fertilizer treatments was low but better than the unfertilized plots. Consequently, the enhanced growth observed in plants treated with combined NPK fertilizer may be due to a better nutrient balance (i.e., N and P). Both N and P are important nutrients that significantly affect plant cellular processes and metabolism. Optimal N supplementation influences plant growth morphology by increasing cytokinin production for cell elongation and the multiplication of meristematic cells [29]. Additionally, N increases plant photosynthetic activity and biomass partitioning between roots and shoots [30]. The study showed that the root biomass of *C. sanguinolenta*, the economic plant component, increased when NPK at 225 kg/ha or N at 200 kg/ha was applied. Such improved production of biomass achieved with fertilizers supplying N confirms previous reports in *Anthriscus cerefolium* [31], *Cymbopogon martini* [27], and *Tagetes minuta* [32].

Meanwhile, P addition is needed for cell division and plant metabolism to ensure maximum plant production [33,34]. The application of P at 250 kg/ha in the present study increased aboveground biomass (109.47 g), yet still, its effect was statistically similar to that obtained by NPK at 262.5 kg/ha (111.68 g) and N at 250 kg/ha (95.10 g) in 12-month-old plants in the field.

The growth response of *C. sanguinolenta* was also influenced by the experimental conditions. For instance, NPK at 262.5 kg/ha recorded the highest aboveground plant biomass in the field, whereas it was low for the same treatment in the pot experiment. These differences may be due to the climatic and growth conditions at the experimental sites: the high rainfall at Bonsu (2.9–140.2 mm) relative to the low rainfall at Legon (14.7–53.6 mm); the location of Bonsu in the forest zone of Ghana, which is the preferred habitat for *C. sanguinolenta* cultivation [35]; limited soil and space in pots for better plant growth.

### 2.2. Effect of Inorganic Fertilizers on Cryptolepine Content

**Figure 1:**
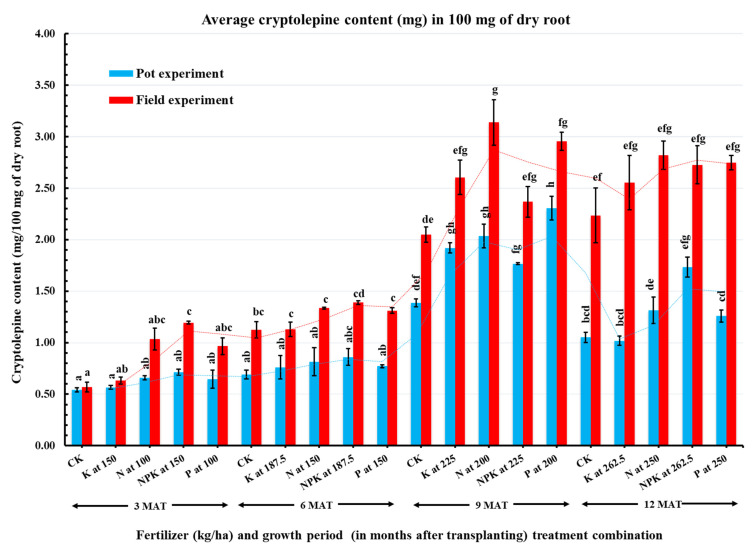
Cryptolepine content in roots of *C. sanguinolenta* in response to fertilizer treatments within four growth periods. The growth periods/plant ages are in months after transplanting (MAT), and CK, K, N, NPK, and P denote the treatments applied: Control, Potassium, Nitrogen, combined NPK, and Phosphorus, respectively. Bars represent the mean of three replicates, and error bars are the SEM. Bars having the same color followed by different letters are significantly different at *p ≤* 0.05 based on the LSD test.

The results show that fertilizer application significantly affected cryptolepine content (mg) in 100 mg of dry root sample compared to unfertilized plants (Figure 1). In the field experiment, the maximum cryptolepine content (3.14 mg) was achieved with the application of N at 200 kg/ha, whereas the least (0.57 mg) was obtained in the unfertilized plants. In the pot experiment, the maximum cryptolepine content was obtained using P at 200 kg/ha (2.30 mg) or N at 200 kg/ha (2.04 mg). The overall cryptolepine content in plants grown in the field was on average 61.4% higher than those grown in pots.

The observed positive effects of nitrogen application on the biosynthesis of the target secondary metabolite in *C. sanguinolenta* confirms previous reports of similar effects in *Datura innoxia* [25], *Catharanthus roseus* L. [24], *Ocimum basilicum* L. [36], *Foeniculum vulgare* Mill. [37], medicinal Pumpkin [38], *Origanum vulgare* L. [39], *Cymbopogon martinii* Roxb. [27] and *Rosmarinus officinalis* L. [40]. One possible explanation for this outcome is that N is a major component in cryptolepine, an indole alkaloid containing nitrogen on its heterocyclic system, and a precursor for proteins and enzymes that play a key role in alkaloid biosynthesis and accumulation in plant roots [41]. Another reason may be attributed to the high rate of N supplied (200 kg/ha) leading to higher photosynthesis, division, and elongation of new cells that are alkaloid secretory ducts and storage glands [42].

Although N (at 200 kg/ha) was the most promising, the results showed that P application enhanced cryptolepine production relative to the unfertilized plants in the 9-month growth period. Ramezani et al. [43] found that the supply of P greatly increased the essential oil content of *O. basilicum.* Similar effects were observed in *Matricaria recutita* by Jeshni et al. [44]. In contrast, the essential oil content in *Lavandula angustifolia* Mill. produced under hydroponic conditions remained unaffected by P supply [45].

### 2.3. Effect of Growth Periods on Cryptolepine Content

Aside from fertilizer application, plant age (in MAT) at harvest significantly influenced the cryptolepine content in the roots (Table 3 and Figure 1). In the field experiment, the cryptolepine content increased with plant age, from 0.88 mg in 3-month-old plants to a peak of 2.63 mg in 9-month-old plants. Cryptolepine content decreased slightly in the 12-month-old plants and was likely the result of senescence at this growth period which reduced the metabolic activity of the plant organs. Similar trends were observed in the pot experiment. The findings on plant age corroborate Amissah et al. [17], who considered 9-month-old *C. sanguinolenta* appropriate for harvesting.

### 2.4. Effect of Treatment Combination (Fertilizers and Growth Periods) on Cryptolepine Yield

**Figure 2:**
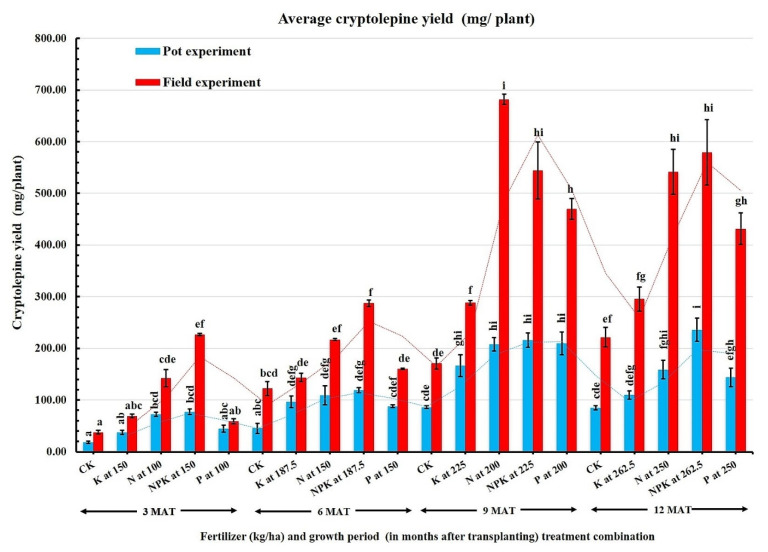
Cryptolepine yield in roots of *C. sanguinolenta* in response to fertilizer treatments within four growth periods. The growth periods/plant ages are in months after transplanting (MAT), and CK, K, N, NPK, and P denote the treatments applied: Control, Potassium, Nitrogen, combined NPK, and Phosphorus, respectively. Bars represent the mean of three replicates, and error bars are the SEM. Bars having the same color followed by different letters are significantly different at *p* ≤ 0.05 based on the LSD test.

Further analysis between the treatments revealed that cryptolepine yields (mg/plant) in both experiments were significantly enhanced by N or NPK treatments at each growth period (Figure 2). The average cryptolepine yield in the field was 284.4 mg and ≈2.5-fold higher than pot-grown plants (116.3 mg) (Table 3). This result highlighted the potential economic gain for producing the herb under field conditions.

Interestingly, the increase in cryptolepine yield paralleled the increase in root biomass in the 9-month-old plants. This was evident in both trials’ significant positive correlation between plant biomass yield and cryptolepine yield (Table 4). Thus, substances that improve root growth and biomass are presumed to increase cryptolepine yield. The economic yield of *C. sanguinolenta* integrates the production of root biomass and cryptolepine synthesis, and both were increased by N fertilization in the present study. As a result, the increase in cryptolepine yield with NPK fertilization could be due to the availability of N in this fertilizer since the root biomass yield for K or P applied alone was relatively low. These findings are consistent with those recorded in Basil, Palmarosa, and Fennel [26,27,28]. They found significant improvement in biomass production and expansion of the leaf area, resulting in greater essential oil yield.

### 2.5. Comparison of Variations in Cryptolepine Yield Due to Fertilizer Application and Growth Period

In both experiments, the nested ANOVA method showed significant variations in cryptolepine yield due to the growth periods and fertilizers applied (Table 5). However, a large variation in the broad sense signifies the factor that is of utmost importance for optimum resource allocation. The variance component study revealed that little variation in cryptolepine yield occurred between fertilizer treatments (33.29% in the field experiment and 29.18% in the pot experiment), as well as between treatment replicates. Much of the observed variations occurred between the growth periods (55.67% in the field and 50.63% in the pot experiments), indicating that growth periods determined the cryptolepine yield even more than fertilizer application. Therefore, plant age must be considered first (9-month cultivation), followed by N fertilizer application for optimum productivity and cryptolepine yield. Our results are consistent with previous reports where several factors, including plant age and nutrition, influenced the yield of alkaloids in different plants [24,46,47,48].

### 2.6. Effects of Inorganic Fertilizer Application on Soil Fertility

The initial soil analysis showed that the soil used in this study had low total N, available K, P, and organic matter content, implying low soil fertility (Table 6). This, therefore, justified the need for amendment with fertilizer to improve the fertility status.

The effect of fertilizer application on the soil macronutrient levels (N, P, and K) in the field and pot experiments at the end of the harvesting periods were compared. In the field experiment, the fertilizers applied significantly affected the macronutrient levels at the end of the harvesting period (Table 7). The highest amount of residual N in the soil after harvesting was obtained when N fertilizer at 100 kg/ha was applied, while the corresponding amount of residual K was obtained when K at 150 kg/ha was applied. Generally, the levels of post-harvest residual N and K decreased after the 3-month growth period. However, in the case of P, application of NPK at 225 kg/ha significantly increased the total P in both the field and pot experiments, compared with the application of P fertilizer alone.

Although the soil nutrients were estimated before planting and after absorption by crops, the influence of fertilizer treatment regimens on soil nutrients (N, P, and K) at the end of the harvesting period may be due to the fertilizers ability to supply soil nutrients readily for use during plant growth and development. These findings agree with studies in which the soil fertility status was improved remarkably by using inorganic fertilizers [22,29,32]. Yousaf et al. [49] noted that P supply and uptake could be optimized when balanced mineral NPK is adopted for soil modification in rapeseed oil cultivation. In the present study, the increase in soil P fraction following the application of NPK rather than P could be due to the synergistic effect of N or K on P mineralization [50]. In addition, the presence of metal oxides (Al and Fe) could influence the adsorption and precipitation of P [51].

In the pot experiment, fertilizer treatments within the same growth period had no significant effect on the levels of macronutrients. This may be due to the extensive use of these supplied nutrients by plants as they mature rapidly in the limited space in the pots.

### 2.7. Effect of Growth Periods on the Bioactivity of C. sanguinolenta Extracts

The bioactivity of cryptolepine from *C. sanguinolenta* plants at different growth periods was tested in vitro against *P. falciparum* strain Dd2. Table 8 shows the concentrations of extracts that can inhibit 50% of plasmodium infected RBCs. The antiplasmodial activity of root extracts from 3-, 6-, and 9-month-old plants in pots was moderately potent (IC_50_ = 6.30–9.23 µg/mL). However, root extracts from 12-month-old plants showed the highest potency (2.56 µg/mL) with about two to three-fold stronger antiplasmodial activity. On the other hand, 9-month-old plants showed the highest potency (4.65 µg/mL) in the field experiment.

Interestingly, the results from both experiments showed increased antiplasmodial activity as the plants aged. This follows the increasing cryptolepine content observed with plant age in the previous experiment (Table 3), indicating the relevance of plant age on the effectiveness of *C. sanguinolenta* decoctions for malaria treatment.

An independent-samples t-test was performed to compare the antiplasmodial activity of extracts obtained in both experiments: field versus pot. There was no significant effect for production medium, t (6) = 0.18, *p* = 0.859, despite the slightly higher antiplasmodial activity of plants in the field (average IC_50_ = 6.07 ± 0.78) than potted plants (IC_50_ = 6.37 ± 1.41). The results suggest that *C. sanguinolenta* can be produced either in the field or in pots without losing antimalarial activity. However, the field environment must be considered for production because it represents the plant’s natural habitat, promotes biomass accumulation, and increases cryptolepine yield based on the present findings.

Furthermore, the concentrations needed to reduce RBC survival by 50% (CC_50_) for all the extracts tested were found to be >10 µg/mL, while that for chloroquine was 0.15 ± 0.00 µg/mL and more toxic to RBCs. All the extract concentrations tested were non-cytotoxic to RBCs and thus exhibited high drug selectivity (Table 8). The high selective index for extracts collected from 9-month-old plants in the field (2.15) and 12-month-old plants in pots (3.91) confirmed that plants cultivated under these two growth conditions were the most effective against *P. falciparum* Dd2.

Aside from antiplasmodial activity, the root extracts were cytotoxic to Jurkat leukaemia cell lines in vitro, with CC_50_ values <30.91 μg/mL for the field and <62.53 μg/mL for the pot samples. Although the impact of plant age on anti-cancer activity was not clear, the findings indicated the possible use of cryptolepine as an agent for cancer management and further strengthened previous reports on other human cancer cells [52,53,54].

## 3. Materials and Methods

### 3.1. Study Sites

Two trials were carried out concurrently from April 2013 to July 2014 to investigate the effects of inorganic fertilizers on *C. sanguinolenta*. The field experiment was set up at the Centre for Scientific and Industrial Research–Plant Genetic Resource Research Institute (CSIR-PGRI) farm located in Bunso (N 5°46′, W 1°1′, 204 m above sea level), a tropical rainforest zone with about 1565 mm rainfall and 25.9 °C temperature annually. The pot experiment was set up at the University of Ghana Research Farm located in Legon (N 5°39′, W 0°11′, 37 m above sea level), a coastal savannah zone with about 809 mm rainfall and 26.6 °C temperature annually. Weather conditions during the study period at Bunso were as follows: rainfall (2.9–140.2 mm), temperature (20.6–34.9 °C), and relative humidity (75.5–87.5%). The conditions at Legon were: rainfall (14.7–53.6 mm), temperature (23.4–32.8 °C), and humidity (73.1–86.0%). The field was initially prepared by ploughing and harrowing before raising mounds of 25 to 35 cm high and spaced at 80 × 80 cm.

The soil used in the study was typically a Haplic Lixosol belonging to the Kokofu soil series of Ghana [55], according to the FAO-UNESCO classification [56]. The soil was analyzed for pH_H2O_ (1:1) and exchangeable cations (Na^+^, Ca^+^, and Mg^2+^) using the ammonium acetate extraction method as outlined by Page et al. [57]. The particle-size distribution was determined according to the method described by Bouyoucos [58]; soil organic carbon (OC) according to Black [59]; total N content of the soil by the Kjeldahl method [60]. The available P was determined using the Lamba 45 spectrophotometer (Perkin Elmer, Inc. USA) after extraction in sodium hydrogen carbonate [61]. The K content was determined using a flame photometer (Jenway PFP7, Cole-Parmer Ltd. Staffordshire, UK) after digestion with ammonium acetate [62]. 

For the pot experiment, topsoil collected from the field research site at Bunso was thoroughly mixed and oven-sterilized at 72 °C for 3 days. Next, 9.6 kg of soil was filled into 10 L plastic pots and spaced at 80 × 80 cm. These were watered and allowed to drain overnight before transplanting.

### 3.2. Transplanting of Seedlings

*C. sanguinolenta* seeds (accession #201 KA), obtained from the CSIR-PGRRI, were sown directly into nursery containers filled with topsoil and watered. Twelve days after sowing, seedlings emerged and were thinned three weeks later. Healthy 12-week-old uniform seedlings were transplanted onto the mounds (600 seedlings, one seedling per mound) and pots (420 seedlings, one seedling per pot) in July 2013. The transplanted seedlings were irrigated regularly for two weeks. Afterward, irrigation was performed as required based on rainfall.

### 3.3. Field Experiment

Four inorganic fertilizers, namely Urea (supplying 46% N), Triple Super Phosphate (46% P_2_O), Muriate of Potash (60% K_2_O), combined NPK (15-15-15%), and a control (without fertilizer) were studied; their nomenclatures are N, P, K, NPK, and CK, respectively. Fertilizers were nested within four plant growth periods (3, 6, 9, and 12 months after transplanting (MAT)), giving 20 experimental treatments in total. Treatments were assigned to plots based on a randomized complete block design with three replicates. Each replicate contained ten plants. The fertilizer application rate was divided equally and added monthly during the growth period using the band placement method at a depth of 5 cm and 10 cm away from the plant (Table 9).

### 3.4. Pot Experiment

The field experiment was repeated using potted plants with minor modifications to the setup: treatments were completely randomized in all units; each replicate contained five plants; fertilizers were spread widely on the soil surface and stirred into the soil using a hand fork. During the growing season, both the field plots and the pots were regularly cleared of weeds.

### 3.5. Plant Growth Measurements, Crop Harvest, and Biomass Analysis

Eight record plants from a treated plot were harvested once plant maturation met a particular growth period (Table 9). All plants in the pot experiment were used as record plants. The number of vines that developed from the main stem was counted, and the girth of two to three vines was measured with a veneer caliper and recorded. Harvesting was performed manually by digging up the plant along with its roots. Subsequently, the root system was detached from the shoot, and the dry biomass recorded after oven drying the samples at 70 °C to a constant weight.

### 3.6. Soil Analysis

Soil analysis was performed to determine the macronutrient levels in the soil. Soil samples were taken at harvest from the top of five mounds and five pots to a depth (≈15–25 cm), using a soil auger (10 cm barrel and 5 cm diameter). The composite sample was thoroughly mixed and then sieved through a 0.25 mm mesh. The total N, P, and K content in the soil sample was determined as previously described (Section 3.1). The experiment was replicated three times (*n* = 3), using a fresh sample each time.

### 3.7. Estimation of Cryptolepine by High-Performance Liquid Chromatography (HPLC)

Of the eight record plants used to assess plant biomass, three roots were set aside and air-dried under a shade at room temperature (25 °C) for 1 month, then ground together into a fine powder. An amount of 100 mg of the ground sample was soaked in absolute ethanol (50 mL) at 25 °C for 24 h and filtered using a Whatman filter paper. Three replicate extractions (n = 3) were made for each treatment. The extracts obtained were evaporated to dryness in a Rotavapor R114 evaporator (Buchi Labortechnik AG, Fawil, Switzerland), then reconstituted in 20 mL absolute ethanol and stored at 4 °C until use.

The cryptolepine content in the extract was analyzed by HPLC at the Department of Chemistry, University of Ghana, Legon, using the Varian 920-LC Analytical HPLC system (Varian Inc., Palo Alto, USA) and a Pursuit C18 (5 µm particle size) column (250 × 4.6 mm id; Varian; Cat. No. 1215–9307). The mobile phase was an HPLC grade methanol/water (*v/v*, 90:9) adjusted to a pH of 2.4 using dichloroacetic acid (DCA). The analytical conditions and methods of the chromatographic assay were performed as described by Amissah et al. [17]. Cryptolepine was quantified with the help of a standardized curve obtained by HPLC, using different concentrations of standard cryptolepine (Sigma-Aldrich Corp., St. Louis, MO, USA) dissolved in absolute ethanol. The results obtained were expressed in mg of cryptolepine content/100 mg of dry ground roots. The economic yield was calculated according to the equation:

Cryptolepine yield (mg plant^−1^) = Dry root biomass (g plant^−1^) × Cryptolepine content (mg/100 mg of ground dry roots) × 1000 (mg/g).(1)

### 3.8. Bioefficacy of Cryptolepine Extracts

The efficacy of the active ingredient (Cryptolepine) in the roots of *C. sanguinolenta* plants harvested at different ages was determined against malaria parasite and Jurkat cells at the Noguchi Memorial Institute for Medical Research, Accra, Ghana.

A chloroquine-resistant strain (Dd2) of *Plasmodium falciparum* was maintained in continuous culture by the modified method of Trager and Jensen [63]; RPMI 1640 medium containing Hepes (*N*-2-hydroxyethyl piperazine-*N*-2-ethane sulfonic acid), and sodium bicarbonate (NaHCO_3_), but without glutamine (Sigma Chemical Co., St. Louis, MO, USA), was used. The medium was supplemented with 10% normal human serum (NHS), 1 mg/mL of l-Glutamine, 1 mg/mL of d-Glucose (Sigma-Aldrich Corp. St. Louis, MO, USA), and 80 μg/mL of gentamicin (Gibco BRL Life Technologies, Paisley, Scotland). After informed consent, volunteers from the blood group O Rh+ donated whole blood for the study. All other chemicals were of analytical grade and obtained from the companies mentioned above.

#### 3.8.1. Antiplasmodial Activity of *C. sanguinolenta* Extracts

The antiplasmodial activity of extracts obtained from unfertilized plants (CK) of each harvest period (3, 6, 9, and 12 MAT) in the field and pot experiments were analyzed in vitro using the SYBR^®^ Green assay, with slight modification [64]. First, dilutions of each of the test samples (0–10 μg/mL) were prepared in RPMI 1640 culture medium (supplemented with L-glutamine, gentamicin, albumax preparation, and glucose). Subsequently, 100 μL of *P. falciparum*-infected RBCs (2.3 × 10^8^ cells/mL) at a parasitemia of 1.5% was added to each well. Chloroquine was used as a positive control. All plates were transferred into a humidified candle jar and incubated at 37 °C for 72 h with 5% CO_2_. Next, a 100 μL of SYBR^®^ Green solution (diluted with lysis buffer) was added to each well and incubated in the dark at room temperature for 2 h. The SYBR^®^ Green fluorescence was measured with a Tecan Infinite M200 microplate reader (Tecan Austria GmbH, Grodig, Austria) with excitation and emission wavelength at 485 and 530 nm, respectively. The extract concentration (IC_50_) causing 50% inhibition of *P. falciparum* growth was obtained from the drug concentration-response curve and the mean IC_50_ determined from experiments performed in triplicate (*n* = 3).

#### 3.8.2. Cytotoxicity of *C. Sanguinolenta* Extracts

A modified version of the tetrazolium-based colourimetric assay [65] was used to test samples for their antiplasmodial activity and toxicity to RBCs. Dilutions of each test sample (0–10 μg/mL) were prepared in appropriate solvents and placed in separate wells of a 96-well microtitre plate in triplicates. Subsequently, 100 mL of *P. falciparum*-infected RBCs (2.3 × 10^5^ cells per ml) at a parasitemia of 1.5% was added to each well. The contributions of plant medicine, parasite culture medium, infected and uninfected RBCs to the optical densities were excluded by setting up control experiments for each of these parameters separately alongside the main experiments. Chloroquine was used as a positive control. All plates were transferred into a candle jar and incubated at 37 °C for 5 days under low levels of O_2_ and CO_2_ after which 2.5 mg/mL MTT (3-(4,5-Dimethylthiazol-2-YI)-2,5-Diphenyltetrazolium Bromide) reagent was added to each well, and the plates incubated again for 2 h. A stopping solution (200 µL of acidified isopropanol) was added to each well to dissolve any formazan formed. The experiment was repeated thrice for each sample concentration. The plates were then kept at room temperature in the dark for 24 h, and the optical densities (OD) read at a wavelength of 570 nm using the Tecan Infinite M200 microplate reader. The percentage protection and survival of RBCs from *P. falciparum* destruction were calculated using the modified formula by Ayisi et al. [65]. The values were plotted against the concentrations of extracts, and the concentration causing 50% cell survival (cytotoxic concentrations, CC_50_) was determined. The selective indices of the extracts were also computed as the ratio of CC_50_ to IC_50_ value.

#### 3.8.3. Anti-Cancer Activity of *C*. *sanguinolenta* Extracts

The anti-cancer properties of the extracts were also evaluated by assessing their cytotoxic effects on human Jurkat cell lines using the MTT assay [65]. Jurkat cell lines were cultured as described by Appiah-Opong et al. [66] and incubated in a humidified chamber at 37 °C and 5% CO_2_. The cells were seeded into a 96 well plate (1× 10^4^ cells/well). Subsequently, the cells were treated with varying extract concentrations (0–100 µg/mL). Curcumin was used as a positive control. After incubating the plate for 72 h, 20 µL of MTT solution (2.5 mg/mL in PBS) was added to each well and further incubated for 4 h. Aliquots of acidified isopropanol (200 µL) were added to each well, and the optical densities were read as described in the previous experiment. The average cytotoxic concentration (CC_50_) was determined from experiments performed in triplicate (*n* = 3).

### 3.9. Data Analysis

Data collected were checked for normality and homogeneity of variances before using the ANOVA test. When the results showed statistical significance (*p*-value ≤ 0.05), heterogeneous group means were separated with the least significant difference test (LSD). A variance component analysis was performed to estimate the relative contribution of growth periods and fertilization to the total variation in cryptolepine yield by nesting the fertilizer treatments within the growth periods. Additionally, the relationship between various plant parameters in the study was calculated using the Pearson correlation index (*r*). Results were presented as means and their standard errors. All statistical tests were carried out at a 5% significance level in Genstat V12 statistical package [67].

## 4. Conclusions

The study showed that mineral fertilization and plant age at harvest positively affect the cryptolepine yield in *C. sanguinolenta*. N application (with or without P and K) increases root biomass yield, cryptolepine content, and cryptolepine yield. The 9-month growth period was confirmed as optimal for harvesting the herb and determines cryptolepine yield even more than fertilizer application. In comparison, field production of the herb improved cryptolepine yield better than production in pots, but this did not significantly change the antiplasmodial activity of cryptolepine extracts, which increased with ageing in the plants.

Based on the present findings, 200 kg/ha of N applied in six equal splits along with a 9-month growth period is recommended for cultivating the herb. Monitoring the active ingredient concentration during growth and maturation to ensure that plants are harvested at the right time with maximum bioactivity should be a major goal of the herbal industry. From the practical point of view, the increase in root biomass and cryptolepine yield induced by N application has positive implications since the commercial value of *C. sanguinolenta* and farmer income depends on the amount of cryptolepine produced.

## Figures and Tables

**Table 1:** Effects of inorganic fertilizers on vine number and girth of *C. sanguinolenta* at different plant growth periods.

Treatment		Field Experiment				Pot Experiment			
Fertilizer (kg/ha)	Plant Age (in MAT)	No of Vines		Vine Girth (cm)		No of Vines		Vine Girth (cm)	
CK	3	2.33 ± 0.33	^a^	2.90 ± 0.35	^abc^	2.33 ± 0.33	^ab^	1.47 ± 0.46	^a^
K at 150	3	3.33 ± 0.33	^abc^	3.27 ± 0.29	^bcd^	2.33 ± 0.33	^ab^	1.57 ± 0.37	^a^
N at 100	3	3.67 ± 0.33	^abcd^	2.49 ± 0.33	^ab^	3.00 ± 0.58	^ab^	2.07 ± 0.34	^abc^
NPK at 150	3	4.33 ± 0.33	^bcd^	3.97 ± 0.17	^cde^	3.67 ± 0.33	^bc^	2.20 ± 0.58	^abc^
P at 100	3	3.00 ± 0.12	^ab^	3.23 ± 0.88	^bcd^	3.33 ± 0.33	^abc^	1.63 ± 0.34	^ab^
CK	6	3.00 ± 0.12	^ab^	2.63 ± 0.36	^abc^	3.67 ± 0.33	^bc^	2.04 ± 0.09	^abc^
K at 187.5	6	3.33 ± 0.33	^abc^	3.54 ± 0.58	^bcd^	4.67 ± 0.33	^cd^	3.79 ± 0.58	^de^
N at 150	6	4.67 ± 0.33	^bcd^	3.64 ± 0.37	^bcd^	5.33 ± 0.33	^d^	3.73 ± 0.58	^de^
NPK at 187.5	6	5.00 ± 0.17	^cd^	4.50 ± 0.66	^de^	7.00 ± 0.58	^e^	3.70 ± 0.58	^de^
P at 150	6	3.67 ± 0.33	^abcd^	2.82 ± 0.29	^abc^	6.00 ± 0.58	^de^	2.81 ± 0.58	^abcde^
CK	9	3.67 ± 0.33	^abcd^	2.70 ± 0.11	^abc^	3.33 ± 0.33	^abc^	2.69 ± 0.46	^abcde^
K at 225	9	3.67 ± 0.33	^abcd^	2.63 ± 0.17	^abc^	4.67 ± 0.88	^cd^	2.45 ± 0.65	^abcd^
N at 200	9	3.33 ± 0.33	^abc^	1.81 ± 0.27	^a^	3.33 ± 0.33	^abc^	2.67 ± 0.12	^abcde^
NPK at 225	9	3.00 ± 0.58	^ab^	2.35 ± 0.23	^ab^	5.33 ± 0.67	^d^	3.88 ± 1.01	^e^
P at 200	9	5.33 ± 0.58	^d^	3.23 ± 0.35	^bcd^	3.00 ± 0.25	^ab^	2.06 ± 0.25	^abc^
CK	12	4.00 ± 1.00	^bcd^	2.58 ± 0.33	^ab^	2.00 ± 0.25	^a^	2.62 ± 0.42	^abcde^
K at 262.5	12	4.00 ± 0.58	^bcd^	2.69 ± 0.20	^abc^	3.00 ± 1.00	^ab^	2.80 ± 0.44	^abcde^
N at 250	12	4.67 ± 1.20	^cd^	2.62 ± 0.06	^abc^	3.67 ± 0.33	^bc^	3.00 ± 0.43	^bcde^
NPK at 262.5	12	5.00 ± 1.53	^cd^	5.15 ± 1.41	^e^	3.67 ± 0.33	^bc^	3.41 ± 0.20	^cde^
P at 250	12	5.33 ± 0.88	^d^	2.96 ± 0.12	^abc^	3.33 ± 0.67	^abc^	3.11 ± 0.19	^cde^

Values represent the average of three replicates ± standard error of the mean (SEM). Treatment means followed by different letters in a column are significantly different at *p* ≤ 0.05 based on the LSD test.

**Table 2:** Effects of inorganic fertilizers on biomass yield of *C. sanguinolenta* at different plant growth periods.

Treatment		Field Experiment						Pot Experiment					
Fertilizer (kg/ha)	Plant Age (in MAT)	Dry Root Biomass (g)		Aboveground Dry Biomass (g)		Shoot/Root Ratio		Dry Root Biomass (g)		Aboveground Dry Biomass (g)		Shoot/Root Ratio	
CK	3	6.63 ± 0.88	^ab^	16.10 ± 0.50	^a^	2.53 ± 0.39	^abcd^	3.33 ± 0.55	^a^	4.90 ± 1.15	^a^	1.44 ± 0.11	^ab^
K at 150	3	10.87 ± 0.41	^bcde^	21.37 ± 0.91	^ab^	1.98 ± 0.14	^ab^	6.54 ± 1.43	^abc^	9.73 ± 0.88	^ab^	1.68 ± 0.50	^abc^
N at 100	3	13.53 ± 0.54	^def^	22.80 ± 1.02	^abc^	1.70 ± 0.14	^a^	11.18 ± 1.85	^defg^	15.57 ± 0.33	^cd^	1.46 ± 0.19	^ab^
NPK at 150	3	18.97 ± 0.38	^ghi^	29.57 ± 1.45	^cd^	1.56 ± 0.07	^a^	10.94 ± 1.80	^defg^	25.03 ± 1.15	^ghi^	2.48 ± 0.59	^cde^
P at 100	3	6.14 ± 0.29	^a^	21.00 ± 0.40	^ab^	3.43 ± 0.12	^bcd^	6.73 ± 0.33	^abc^	12.07 ± 1.20	^bc^	1.81 ± 0.22	^abcd^
CK	6	10.60 ± 1.01	^bcd^	27.73 ± 0.60	^bcd^	2.66 ± 0.23	^abcd^	6.07 ± 1.98	^ab^	9.53 ± 1.72	^ab^	1.71 ± 0.21	^abc^
K at 187.5	6	12.73 ± 0.19	^cdef^	35.00 ± 1.25	^def^	2.75 ± 0.14	^abcd^	13.37 ± 1.85	^fg^	15.8 ± 0.50	^cd^	1.18 ± 0.11	^a^
N at 150	6	16.27 ± 0.29	^fgh^	40.97 ± 0.87	^ef^	2.52 ± 0.01	^abcd^	13.73 ± 0.24	^fg^	25.47 ± 0.39	^ghi^	1.91 ± 0.06	^abcd^
NPK at 187.5	6	20.67 ± 0.47	^i^	44.00 ± 0.87	^f^	2.13 ± 0.01	^abc^	14.40 ± 1.73	^g^	41.47 ± 0.94	^k^	2.95 ± 0.29	^e^
P at 150	6	12.27 ± 0.70	^cdef^	31.63 ± 0.95	^de^	2.60 ± 0.18	^abcd^	11.40 ± 0.62	^defg^	16.57 ± 0.45	^cde^	1.46 ± 0.04	^ab^
CK	9	8.56 ± 1.66	^abc^	44.75 ± 4.39	^fg^	5.43 ± 0.52	^f^	6.23 ± 0.50	^ab^	15.85 ± 1.18	^cd^	2.55 ± 0.10	^cde^
K at 225	9	11.50 ± 1.80	^cde^	61.11 ± 8.62	^h^	5.44 ± 0.47	^f^	8.39 ± 1.18	^bcde^	19.32 ± 2.38	^def^	2.43 ± 0.13	^cde^
N at 200	9	22.40 ± 2.50	^i^	75.88 ± 4.66	^i^	3.45 ± 0.61	^bcd^	10.22 ± 1.39	^cdef^	23.77 ± 2.61	^fgh^	2.42 ± 0.08	^cde^
NPK at 225	9	22.68 ± 2.88	^i^	77.67 ± 5.79	^i^	3.52 ± 0.33	^cd^	12.17 ± 1.20	^efg^	25.80 ± 1.18	^ghi^	2.14 ± 0.45	^bcde^
P at 200	9	16.07 ± 1.91	^fgh^	60.19 ± 1.45	^h^	3.89 ± 0.81	^de^	8.91 ± 0.97	^bcde^	21.74 ± 1.59	^fg^	2.46 ± 0.58	^cde^
CK	12	10.45 ± 1.17	^abcd^	56.00 ± 0.40	^gh^	5.57 ± 0.92	^f^	8.19 ± 0.98	^bcd^	21.55 ± 2.23	^efg^	2.66 ± 0.23	^de^
K at 262.5	12	12.12 ± 1.63	^cdef^	74.47 ± 8.11	^i^	6.51 ± 0.29	^f^	10.76 ± 1.11	^defg^	23.98 ± 2.82	^fghi^	2.22 ± 0.35	^bcde^
N at 250	12	14.98 ± 1.84	^efg^	95.10 ± 5.23	^j^	6.52 ± 0.67	^f^	12.02 ± 1.30	^defg^	28.38 ± 3.33	^hij^	2.46 ± 0.46	^cde^
NPK at 262.5	12	21.01 ± 2.77	^i^	111.68 ± 12.24	^j^	5.36 ± 0.73	^ef^	13.48 ± 1.88	^fg^	31.27 ± 2.12	^j^	2.41 ± 0.52	^cde^
P at 250	12	19.59 ± 1.44	^hi^	109.47 ± 7.10	^j^	5.68 ± 1.38	^f^	11.12 ± 1.68	^defg^	29.10 ± 2.95	^ij^	2.72 ± 0.06	^de^

Values represent the average of three replicates ± SEM. Treatment means followed by different letters in a column are significantly different at *p* ≤ 0.05 based on the LSD test.

**Table 3:** Mean cryptolepine content and yield at different harvest periods.

Plant Age at Harvest (in MAT)	Field Experiment		Pot Experiment	
	Cryptolepine Content (mg) in 100 mg of Dry Roots	Cryptolepine Yield (mg/plant)	Cryptolepine Content (mg) in 100 mg of Dry Roots	Cryptolepine Yield (mg/plant)
3	0.88 ± 0.14 ^a^	106.70 ± 33.86 ^a^	0.63 ± 0.07 ^a^	49.94 ± 11.98 ^a^
6	1.26 ± 0.08 ^b^	186.00 ± 29.33 ^a^	0.78 ± 0.12 ^a^	91.50 ± 18.56 ^b^
9	2.63 ± 0.27 ^c^	431.06 ± 91.36 ^b^	1.88 ± 0.17 ^c^	177.10 ± 30.95 ^c^
12	2.61 ± 0.28 ^c^	413.90 ± 81.98 ^b^	1.28 ± 0.16 ^b^	146.60 ± 31.44 ^c^
mean	1.84 ± 0.19	284.39 ± 42.91	1.14 ± 0.12	116.26 ± 14.42

Values represent the average of five replicates ± SEM. Treatment means followed by different letters in a column are significantly different at *p* ≤ 0.05 based on the LSD test.

**Table 4:** Correlation between plant biomass, vine number, girth, cryptolepine content and yield in *C. sanguinolenta*.

	Root Biomass	Shoot Biomass	Vine Number	Vine Girth	Cryptolepine Content	Cryptolepine Yield
Root biomass		0.78 **	0.57 **	0.83 **	**0.16**	0.53 *
Shoot biomass	0.61 **		0.46 *	0.71 **	**0.39**	0.65 **
Vine number	0.53 *	0.57 **		0.65 **	**0.01**	**0.19**
Vine girth	0.25	**0.05**	0.49 *		**0.25**	0.54 *
Cryptolepine content	0.50 *	0.88 **	0.50 *	−0.20		0.89 **
Cryptolepine yield	0.82 **	0.88 **	0.50 *	−0.03	0.87 **	

* and ** show a significant correlation at alpha levels 5 and 1%, respectively. The non-bold-faced and bold-faced coefficients show correlations in the field and pot experiments, respectively.

**Table 5:** An estimate of variance due to growth periods and fertilizer treatments on cryptolepine yield in the field and pot experiments.

Source of Variation	Degrees of Freedom	Sum of Squares	Mean Squares	*F*-Value	*p*-Value	Variance Component	% of Total
Field Experiment							
Growth period (GP)	3	1167.58	389.19	8.53	0.001	22.90	55.67
Block	2	3.44	1.72	0.37	0.696	0.00	0.00
Fertilizer (GP)	16	730.07	45.63	9.72	0.000	13.70	33.29
Error	38	178.32	4.69			4.54	11.04
Total	59	2079.41				41.14	100.00
Pot Experiment							
Growth period (GP)	3	347.20	115.73	9.26	0.001	6.76	50.63
Fertilizer (GP)	16	200.10	12.50	3.61	0.000	3.90	29.18
Error	40	138.40	3.46			2.70	20.19
Total	59	685.70				13.36	100.00

Variance component was estimated using the nested method of ANOVA: fertilizer treatments were nested within the growth periods, *p*-values < 0.05 are significant at 5% alpha level.

**Table 6:** Physical and chemical characteristics of soil at 0–20 cm depth before planting.

pH(H_2_O)	Avail. P (mg/kg)	% N	% OC	Exchangeable Cations (cmol/kg)				Particle Size (%)		
				K^+^	Na^+^	Ca^2+^	Mg^2+^	Sand	Silt	Clay
5.50	9.72	0.09	0.60	0.14	0.70	2.60	2.00	52.90	22.10	25.00

**Table 7:** Effects of inorganic fertilizer application on soil macronutrients (N, P, and K) levels at the end of the different harvesting periods.

Treatment		Field Experiment						Pot Experiment					
Fertilizer (kg/ha)	Plant Age (months)	Total N (%)		P (mg/kg)		K (cmol/kg)		Total N (%)		P (mg/kg)		K (cmol/kg)	
CK	3	0.84 ± 0.37	^b^	9.62 ± 0.58	^ab^	1.10 ± 0.58	^cd^	1.40 ± 0.20	^b^	17.54 ± 0.58	^a^	1.50 ± 0.33	^b^
K at 150	3	1.42 ± 0.33	^c^	29.77 ± 0.66	^f^	3.02 ± 0.33	^f^	1.64 ± 0.39	^b^	16.17 ± 0.58	^a^	1.71 ± 0.33	^b^
N at 100	3	1.93 ± 0.42	^d^	16.29 ± 0.61	^bcd^	1.09 ± 0.58	^bcd^	1.68 ± 0.31	^b^	16.36 ± 0.58	^a^	1.46 ± 0.33	^b^
NPK at 150	3	1.15 ± 0.58	^cd^	72.50 ± 0.58	^i^	2.12 ± 0.37	^e^	1.43 ± 0.33	^b^	65.28 ± 0.58	^g^	1.69 ± 0.33	^b^
P at 100	3	1.50 ± 0.30	^cd^	39.24 ± 0.58	^g^	1.48 ± 0.30	^d^	1.34 ± 0.33	^b^	42.02 ± 0.58	^cd^	1.46 ± 0.33	^b^
CK	6	0.07 ± 0.00	^a^	29.00 ± 0.73	^ef^	0.10 ± 0.01	^a^	0.07 ± 0.03	^a^	14.43 ± 0.38	^a^	0.13 ± 0.00	^a^
K at 187.5	6	0.06 ± 0.00	^a^	19.78 ± 0.85	^cde^	0.50 ± 0.02	^ab^	0.07 ± 0.01	^a^	14.49 ± 1.31	^a^	0.31 ± 0.01	^a^
N at 150	6	0.07 ± 0.00	^a^	25.11 ± 0.83	^def^	0.09 ± 0.01	^a^	0.07 ± 0.00	^a^	12.20 ± 0.23	^a^	0.11 ± 0.10	^a^
NPK at 187.5	6	0.09 ± 0.00	^a^	54.51 ± 1.06	^h^	0.33 ± 0.02	^a^	0.09 ± 0.00	^a^	56.03 ± 2.63	^ef^	0.19 ± 0.01	^a^
P at 150	6	0.06 ± 0.00	^a^	75.13 ± 4.07	^i^	0.10 ± 0.01	^a^	0.06 ± 0.03	^a^	34.90 ± 1.07	^bc^	0.13 ± 0.00	^a^
CK	9	0.16 ± 0.01	^a^	8.03 ± 0.64	^ab^	0.09 ± 0.03	^a^	0.10 ± 0.01	^a^	17.87 ± 1.34	^a^	0.15 ± 0.01	^a^
K at 225	9	0.13 ± 0.01	^a^	6.72 ± 1.93	^a^	0.55 ± 0.05	^abc^	0.09 ± 0.01	^a^	48.80 ± 5.21	^de^	0.20 ± 0.03	^a^
N at 200	9	0.12 ± 0.02	^a^	7.78 ± 1.49	^ab^	0.09 ± 0.01	^a^	0.10 ± 0.03	^a^	54.31 ± 5.71	^ef^	0.13 ± 0.01	^a^
NPK at 225	9	0.15 ± 0.01	^a^	93.35 ± 1.70	^j^	0.44 ± 0.05	^a^	0.09 ± 0.00	^a^	76.68 ± 3.02	^h^	0.20 ± 0.01	^a^
P at 200	9	0.13 ± 0.02	^a^	70.40 ± 12.93	^i^	0.09 ± 0.01	^a^	0.10 ± 0.01	^a^	57.20 ± 5.09	^f^	0.14 ± 0.01	^a^
CK	12	0.12 ± 0.01	^a^	13.10 ± 1.31	^abc^	0.05 ± 0.01	^a^	0.15 ± 0.01	^a^	11.97 ± 1.06	^a^	0.05 ± 0.03	^a^
K at 262.5	12	0.12 ± 0.01	^a^	16.36 ± 1.81	^bcd^	0.35 ± 0.07	^a^	0.15 ± 0.00	^a^	10.55 ± 0.91	^a^	0.15 ± 0.02	^a^
N at 250	12	0.12 ± 0.01	^a^	9.32 ± 0.85	^ab^	0.06 ± 0.00	^a^	0.20 ± 0.01	^a^	14.31 ± 1.39	^a^	0.07 ± 0.01	^a^
NPK at 262.5	12	0.09 ± 0.01	^a^	43.20 ± 1.12	^g^	0.12 ± 0.01	^a^	0.16 ± 0.01	^a^	51.53 ± 3.77	^ef^	0.08 ± 0.03	^a^
P at 250	12	0.10 ± 0.00	^a^	20.12 ± 1.72	^cde^	0.07 ± 0.01	^a^	0.16 ± 0.03	^a^	31.15 ± 3.17	^b^	0.06 ± 0.01	^a^

Values represent the average of three replicates ± SEM. Treatment means followed by different letters in a column are significantly different at *p ≤* 0.05 based on the LSD test.

**Table 8:** The effects of plant growth period on the antiplasmodial and anti-cancer activity of *C. sanguinolenta* extracts.

Growth Period (in MAT)	Antiplasmodial Activity		Antiplasmodial Selectivity Index (SI)		Anti-Cancer Activity (Cell Viability)	
	IC_50_ Values for Pot Expt. (µg/mL)	IC_50_ Values for Field Expt. (µg/mL)	SI for Pot Expt. (*CC_50_/IC_50_)	SI for Field Expt. (*CC_50_/IC_50_)	CC_50_ Values for Pot Expt. (µg/mL)	CC_50_ Values for Field Expt. (µg/mL)
3	9.23 ± 0.05	8.12 ± 0.07	1.08	1.23	6.83 ± 1.03	12.25 ± 1.41
6	7.37 ± 0.06	6.44 ± 0.56	1.36	1.55	30.91 ± 1.95	8.81 ± 0.76
9	6.30 ± 0.30	4.65 ± 0.02	1.59	2.15	11.7 ± 2.52	8.68 ± 0.46
12	2.56 ± 0.04	5.06 ± 1.28	3.91	1.98	–	62.53 ± 6.68
Control	3.01 ± 0.03 ^x^	3.01 ± 0.03 ^x^	0.05	0.05	8.56 ± 1.67 ^y^	8.56 ± 1.67 ^y^

* Because the 50% cytotoxic concentrations (CC_50_) for all extracts were outside the range of extract concentrations tested, the highest sample concentration (10 μg/mL) was considered as the CC_50_ for calculating the selectivity index (SI). ^x^ and ^y^ represent values from controls: chloroquine and curcumin, respectively. Missing data (–).

**Table 9:** Treatments and fertilizer rates used in the field and pot experiment.

Fertilizer	Fertilizer Rates (kg/ha) Used in Each Growth Period (Months after Transplanting)			
	3 Months ^2^*	6 Months ^4^	9 Months ^6^	12 Months ^8^
Urea (N)	100.0	150.0	200.0	250.0
Triple Super Phosphate (P)	100.0	150.0	200.0	250.0
Muriate of Potash (K)	150.0	187.5	225.0	262.5
NPK	150.0	187.5	225.0	262.5
Control (CK)	0.0	0.0	0.0	0.0

* Number of times fertilizer (split dose) was combined within each growth period at monthly intervals.

## Data Availability

Data included in article/referenced in the article.
